# Feasibility, Mechanical Properties, and Environmental Impact of 3D-Printed Mortar Incorporating Recycled Fine Aggregates from Decoration and Renovation Waste

**DOI:** 10.3390/ma19081618

**Published:** 2026-04-17

**Authors:** Pu Yuan, Xinjie Wang, Jie Huang, Quanbin Shi, Minqi Hua

**Affiliations:** 1 School of Urban Construction, Changzhou University, Changzhou 213164, China; yuanpucczuedu@163.com; 2 School of Urban and Rural Construction, Taizhou Polytechnic College, Taizhou 225300, China; 3 School of Civil Engineering & Architecture, Wuhan University of Technology, Wuhan 430070, China

**Keywords:** waste from decoration, 3D printing, section modulus, interlayer bond strength, microhardness

## Abstract

To address the accumulation of construction and demolition waste (W&D), this study recycled it into regenerated fine aggregate and prepared 3D-printed mortars with replacement ratios ranging from 0% to 100%. The mechanical properties of hardened specimens were tested, and the degradation mechanisms of mechanical performance were investigated through SEM, MIP, and microhardness analysis. The carbon emissions of the materials were evaluated. The results indicated that while the 3D-printed mortar exhibited excellent buildability, its compressive strength, flexural strength, and interlayer bond strength gradually decreased with increasing replacement ratio. MIP results showed that as the replacement ratio of the W&D increased from 0% to 100%, the total porosity of the 3D-printed specimens significantly increased from 14.7433% to 27.5903%. SEM and microhardness images confirmed severe ITZ deterioration, and the average ITZ width increased from 31 to 79 μm. As the W&D replacement ratio increased from 0% to 100%, the total GWP decreased from 0.4043 to 0.3800 kg CO_2_-eq/kg mortar. Maximizing the utilization of W&D is key to achieving efficient utilization of solid waste. Considering printability, mechanical performance, interlayer behavior, microstructural characteristics, and environmental impact in a comprehensive manner, the 80% W&D replacement ratio can be regarded as a relatively balanced and promising selection. This work not only suggests the technical feasibility of recycling W&D in 3D printing mortar, but also proposes a sustainable pathway to reduce carbon emissions in construction.

## 1. Introduction

In recent years, a consistent decline in domestic demand for natural sand and gravel has been observed, driven by the deceleration of infrastructure construction in China and a significantly heightened public awareness of ecological conservation and sustainable resource utilization. Moreover, the extraction of river sand has been demonstrated to cause severe adverse environmental impacts [1,2], necessitating the exploration of more waste resources as alternative materials. An increase in the generation of construction waste from decoration and renovation (W&D) has been witnessed [3]. Frequent and high-standard renovation activities, while creating ideal living spaces, have simultaneously resulted in greater volumes of W&D. Furthermore, a considerable amount of W&D has been contributed by the renovation and demolition of old buildings [4,5]. However, current recycling pathways for W&D remain limited. W&D is typically transported to suburban areas for landfill or accumulation, which occupies extensive land resources and poses potential environmental threats [6].

Under this context, the recycling and reutilization of W&D has been recognized as an effective approach to repurpose demolished materials and address environmental pollution [7,8,9,10]. Building 3D-printing technology [11] has attracted extensive attention from both academia and engineering sectors due to its remarkable advantages in enabling architectural design flexibility, reducing formwork and labor requirements, and enhancing construction automation [12]. Essentially, this technology constitutes a digital model-based “additive construction” process in which structures are fabricated through layer-by-layer material deposition [13,14]. This method allows digital designs to be directly transformed into physical buildings, thereby eliminating the constraints imposed by traditional formwork on architectural morphology. As a result, complex, lightweight, and highly efficient structural forms [10,15,16] generated through parametric design and topological optimization [17] can be realized at low cost. Although the concept of 3D printing was initially proposed as early as the 1990s [18,19,20] and research on concrete-based printing technologies has been ongoing for over two decades, significant challenges remain in the design of complex material systems and the construction of non-standardized structural forms [21].

More importantly, 3D-printing technology creates a substantial and continuous demand for printing materials, particularly sand as the core aggregate. Currently, research on the processing and performance of natural aggregates in 3D printing has reached a relatively mature stage [22,23]. For instance Lyu et al. [24] proposed a novel multilayered plant-growth concrete based on aggregate-bed 3D printing, designed to support vegetation. Concurrently, investigations into other types of construction and demolition waste, such as recycled sand [25,26,27], are gradually emerging. Hou et al. [28] partially replaced cement (10%, 20%, 30%) with recycled powder to produce mortar for 3D printing, observing effective improvements in early-age mechanical properties and buildability. Pasupathy et al. [29] studied the influence of brick waste content on the fresh properties of printable mixtures, noting that the incorporation of brick waste reduced flowability, but enhanced compressive strength and reduced carbon emissions by 60–80%. Mai et al. [30] employed large-particle (36 mm) recycled aggregates as a particle bed and applied shotcrete for 3D concrete printing, demonstrating that the use of coarse aggregates could reduce the cement volume fraction by more than 50% compared to conventional mortar-based printing.

Although several studies have explored the use of recycled aggregates or recycled powder from construction and demolition waste in 3D-printing materials, these studies have mainly focused on relatively homogeneous waste streams. Materials such as construction waste [31], waste rubber [32], and waste glass [33] have been partially validated as feasible for printing, showing potential in non-load-bearing components and landscape wall applications [34,35]. In contrast, there is limited application of decoration and renovation waste in 3D-printable cementitious materials. W&D is much more heterogeneous and often contains aged plaster, tile fragments, gypsum board residues, wood chips, plastics, and other lightweight impurities. Such compositional complexity may significantly affect the rheological behavior, printing stability, interlayer bonding, and hardened performance of 3D-printed mortar. A systematic understanding of W&D in printable cementitious materials is still lacking.

This study investigates the feasibility of using recycled fine aggregate derived from W&D as a partial or full replacement for natural sand in 3D-printed mortar. The effects of W&D replacement ratio on flowability, extrudability, and buildability were first evaluated. The anisotropic mechanical properties of the printed mortar, including compressive strength, flexural strength, and interlayer bond strength, were then systematically assessed. In addition, SEM, MIP, and microhardness analyses were carried out to reveal the degradation mechanism of the interfacial transition zone and pore structure. Finally, the environmental impact of the developed mixtures was evaluated through carbon emission analysis.

## 2. Materials and Methods

### 2.1. Materials

**Figure 1:**
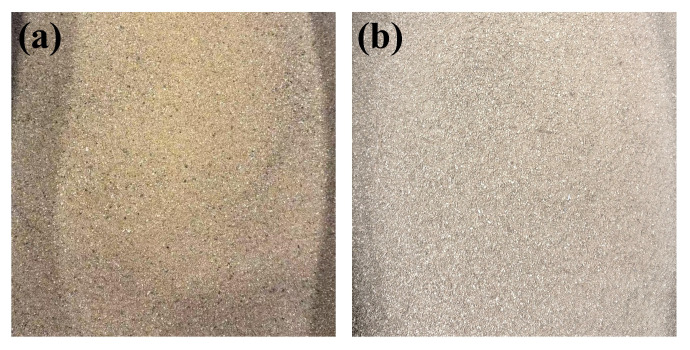
Physical images: (**a**) NS, (**b**) W&D.

Portland cement (grade P·O 42.5, chemical composition provided in Table 1) was used in this experiment. Natural sand (NS) and W&D were employed as fine aggregates, both of which were sieved to a particle size range of 0.075–1.18 mm. Their mechanical properties and photographs are presented in Table 2 and Figure 1, respectively. Compared with NS, W&D exhibited lower apparent density, but much higher water absorption and crushing value, indicating a more porous and weaker aggregate skeleton. This study employed superplasticizer (SP), sodium gluconate (SG), and silica fume (SF) in the mixture. The composition of the SF is shown in Table 3. Laboratory water from Changzhou University was used. Information on all materials is provided in Table 4.

**Figure 2:**
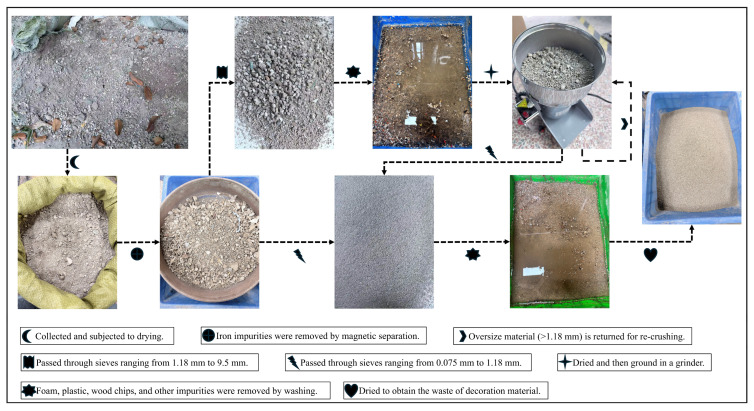
Screening process of W&D.

Unlike construction and demolition waste, renovation waste is composed of a significant amount of discarded interior wall plaster, waste tiles, discarded gypsum boards, and discarded pipes, as well as wood waste and plastics generated during construction. It is characterized by complex composition and a large proportion of lightweight materials. This sorted material underwent successive crushing and washing processes to produce the aggregate used in the experiment. A sieving and preparation flowchart is provided in Figure 2. The utilization rate of W&D from decoration and renovation waste reached 94.7% through a two-stage screening and multi-cycle crushing process.

**Figure 3:**
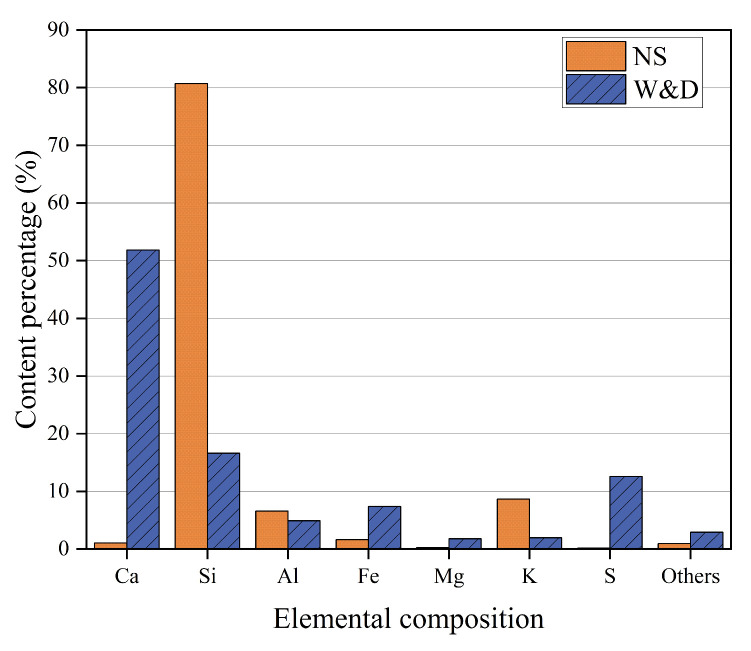
Elemental composition of NS and W&D.

**Figure 4:**
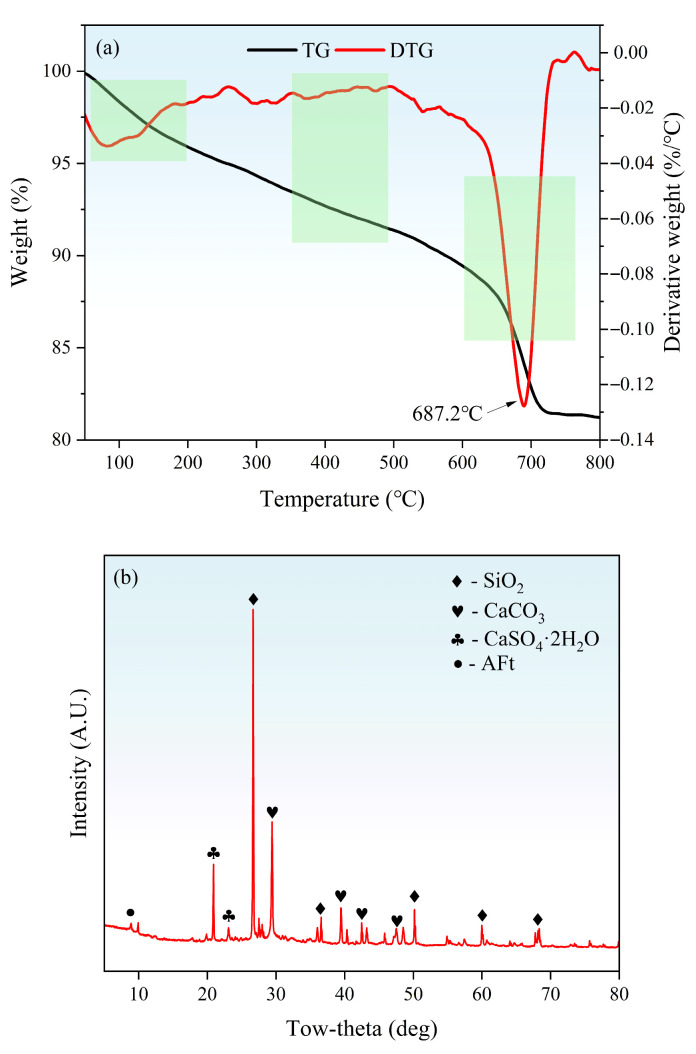
Microscopic analysis of W&D: (**a**) TG, (**b**) XRD.

Figure 3 presents the chemical compositions of the two sands, determined by X-ray fluorescence (XRF) analysis. To determine the specific crystalline phases in W&D, a series of analyses including thermogravimetric analysis (TG), X-ray diffraction (XRD), and scanning electron microscopy (SEM), were conducted. The XRF results indicated significantly higher Ca content in W&D compared to the NS, whereas the Si content exhibited the opposite trend. The phase characteristics were further elucidated by TG and XRD analyses. In Figure 4a, a distinct mass loss was observed around 700 °C, which is attributed to the decomposition of CaCO_3_. In contrast, no significant mass loss step corresponding to Ca (OH)_2_ decomposition was detected around 400 °C. This observation is consistent with the XRD patterns (in Figure 4b), which identified SiO_2_, CaCO_3_, and CaSO_4_·2H_2_O as the primary crystalline phases present in W&D.

**Figure 5:**
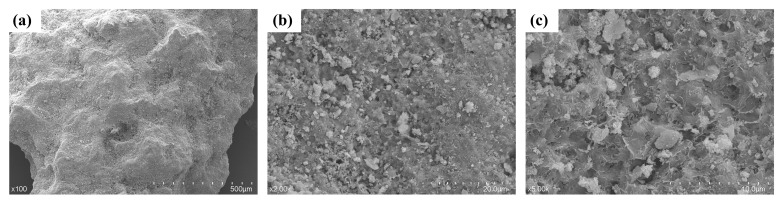
SEM analysis of W&D: (**a**) ×100, (**b**) ×2.00 K, (**c**) ×5.00 K.

Figure 5 shows the particle morphology of W&D and the aged paste attached to its surface directly revealed by SEM observations, providing morphological evidence for the phase analysis above. In Figure 5a, the W&D particles exhibited characteristic rough surfaces and irregular, polygonal geometries, clearly indicating their origin from mechanical crushing processes. In Figure 5b, aged cement paste and unhydrated cement particles were observed to be firmly adhered to the aggregate surface, accompanied by distinct microcracks and pores. In Figure 5c, the hydration products (such as honeycombed C-S-H gel, layered portlandite, and needle-shaped ettringite) form a loose and porous microstructure, with visible microcrack propagation. These defects were identified as the primary factors contributing to the high water absorption and performance degradation of W&D.

### 2.2. Mix Proportion Design

Table 5 lists the six mix designs prepared with a constant sand-to-cement ratio of 1.0, where the content of W&D was set at 0%, 20%, 40%, 60%, 80%, and 100% by sand weight percentage. The content of other materials was unchanged. SF was incorporated at 5% of the cement, with SG and SP dosages fixed at 0.6% and 0.5%, respectively. Because W&D exhibits high water absorption, its water demand increased with dosage. To ensure flowability and printability, supplementary water was determined based on preliminary experiments. This was set as the amount corresponding to an initial mean flow diameter of the cement mortar exceeding 180 mm. W&D-xx% denotes the mortar in which xx% of natural sand (by mass) was replaced by recycled fine aggregate from decoration and renovation waste.

### 2.3. Printing System and Specimen Preparation

**Figure 6:**
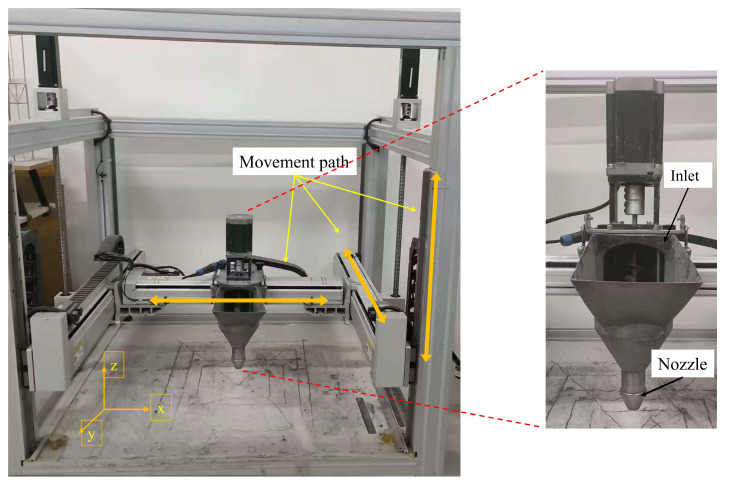
3D printer.

The 3D printer manufactured by Hangzhou Guanli Intelligent Technology Co., Ltd. (Hangzhou, China) is a gantry-type 3D printer, as shown in Figure 6. The machine dimensions are 1280 mm × 1150 mm × 1400 mm, and the maximum printable dimensions are 500 mm × 700 mm × 450 mm. In order to avoid the influence of the printing process parameters, during the printing process, the nozzle travel speed was set to 50 mm/s, and after continuous adjustment, the extrusion speed was determined as 1.5 r/s. The extrusion width was consistent with the nozzle width of 20 mm, and the layer height was set to 10 mm. The length of the 3D-printed specimens was 175 mm, with a printing accuracy of 1 mm.

In accordance with the standard GB/T 17671-2021 [36] testing method for cement mortar strength (ISO method), the testing environment was maintained at a relative humidity of 60% ± 5% and a temperature of 20 ± 2 °C. The mortar mixing procedure was as follows. Water, SP, and SG were added to the mixing pot, followed by cement and SF. The pot was then fixed onto the mixer and raised to its working position. The machine started immediately, mixing at low speed for 30 ± 1 s. Subsequently, at the beginning of the second 30 ± 1 s period, sand was added uniformly. The mixer was then switched to high speed and mixed for a further 30 ± 1 s. After mixing, it was then stopped for 90 s. During the first 15 ± 1 s of this interval, the bowl was lowered, and the mortar adhering to the blades, bowl wall, and bottom was scraped off into the bowl using a trowel. Mixing was then resumed at high speed for 60 ± 1 s. The mixer was stopped, and the mixed mortar was subsequently used to fabricate the specimens.

For cast specimens, two types of standard specimens were prepared: prismatic specimens with dimensions of 40 mm × 40 mm × 160 mm and cubic specimens with dimensions of 40 mm × 40 mm × 40 mm. Three specimens were fabricated for each mix proportion. The 3D-printed specimens, after initial setting for 24 h, along with the cast specimens, were placed in a standard curing room with a temperature of 20 ± 1 °C and relative humidity of 90% or above for curing ages of 7 days and 28 days.

### 2.4. Test Methods

#### 2.4.1. Printability Test Methods

**Figure 7:**
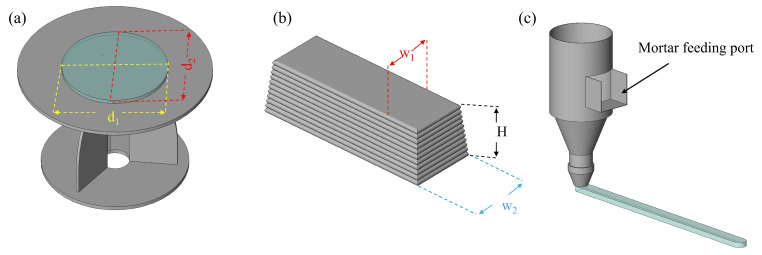
Printability test methods: (**a**) flowability, (**b**) buildability, (**c**) extrudability.

As shown in Figure 7a, the flowability was measured and recorded every 10 min over a period of 2 h with reference to the standard GB/T 2419-2005 [37] testing method for fluidity of cement mortar. Prior to testing, the mortar was poured into the mold. After 25 consecutive jolts, two values of the mortar diameter, *d*_1_ and *d*_2_, were recorded and their average was taken as the flowability value. Good flowability ensures that the mortar can be transported from the printing funnel to the nozzle [38].

Buildability refers to the ability of printed mortar to resist deformation and maintain its structural integrity while supporting the loads from upper layers [39,40]. In this study, the horizontal distance between the top and bottom surfaces of the 12-layer specimen after 28 days of curing, as well as the vertical height of the specimen, were measured. The ratio of the horizontal displacement to the vertical height, expressed as tanθ, was then calculated to evaluate the buildability [41] of the printed mortar, as illustrated in Figure 7b. which can be calculated using Equation (1):

(1) $$\tan\theta =\frac{W_{2}-W_{1}}{2H}$$

where *H* represents the height of the specimen, *W*_2_ represents the bottom width, and *W*_1_ represents the top width. tanθ values were recorded for each specimen, and the mean and standard deviation were calculated for each group. A smaller tanθ value indicates better buildability of the specimen.

Extrudability was evaluated by observing and measuring the average width of the printed filament and by examining the filament surface for fractures or defects [42], as illustrated in Figure 7c.

#### 2.4.2. Mechanical Strength Test Methods

**Figure 8:**
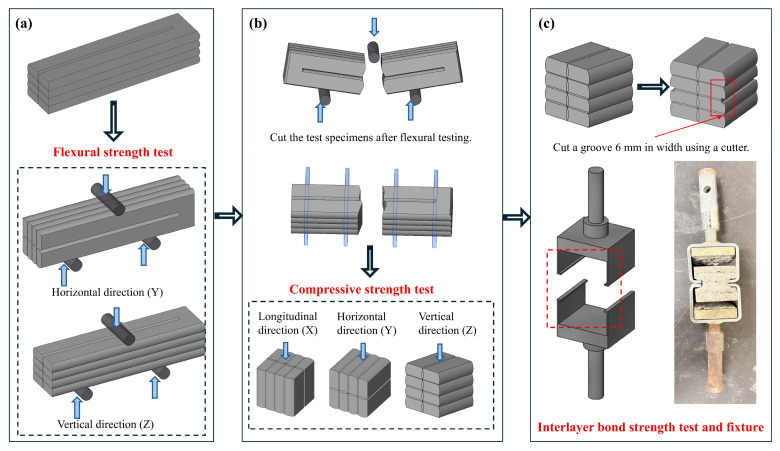
Mechanical strength test methods: (**a**) flexural strength (**b**) compressive strength (**c**) interlayer bond strength.

The printing travel direction is designated as the X-axis, the direction perpendicular to the printing path as the Y-axis, and the direction normal to the XY-plane as the Z-axis. Figure 8a shows the 3D-printed specimens for flexural strength tests. The flexural strengths along the Y and Z directions were recorded separately. Cubic specimens were prepared for compressive strength tests. These cubic specimens were subjected to compressive loading along the X, Y, and Z directions, and the corresponding compressive strengths were recorded, as detailed in Figure 8b. For each test, three specimens were used, and the average value of the test data was calculated as the average strength. All mechanical property tests were conducted in accordance with the standard GB/T 17671-2021.

During the interlayer bond strength test, steel plate molds with a thickness of 10 mm and a side length of 40 mm featuring 25 mm-thick L-shaped extensions on both sides were placed on the upper and lower surfaces of the specimen (Figure 8c). Grooves with a depth of 3 mm and a thickness of 6 mm were made on both sides of the specimen to engage with the corresponding slots in the L-shaped parts of the molds. The average width (b) and length (l) of the fractured interlayer contact surface were measured, and the fracture contact area (A) was calculated as A=b×l. Six samples were tested, and the average value was taken. The interlayer bond strength of the concrete was calculated using Equation (2):

(2) f=F/A

where f is the splitting tensile strength of concrete (MPa), F denotes the failure load (N), and A represents the interlayer contact area (mm^2^), calculated as A=b×l.

#### 2.4.3. MIP and SEM

Pore size distribution and porosity were measured using mercury intrusion porosimetry. The morphological characteristics of the 3D-printed specimens were observed using an ultrahigh-resolution field emission scanning electron microscope, which offers an adjustable magnification range from 20× to 1,000,000×. The specimens for SEM and MIP analysis had dimensions of less than 15 mm in both length and width and less than 5 mm in height. The test samples for SEM, MIP, and microhardness were all extracted from the region between the fifth and sixth layers of the 12-layer specimen after 28 days of curing.

#### 2.4.4. Microhardness Test

**Figure 9:**
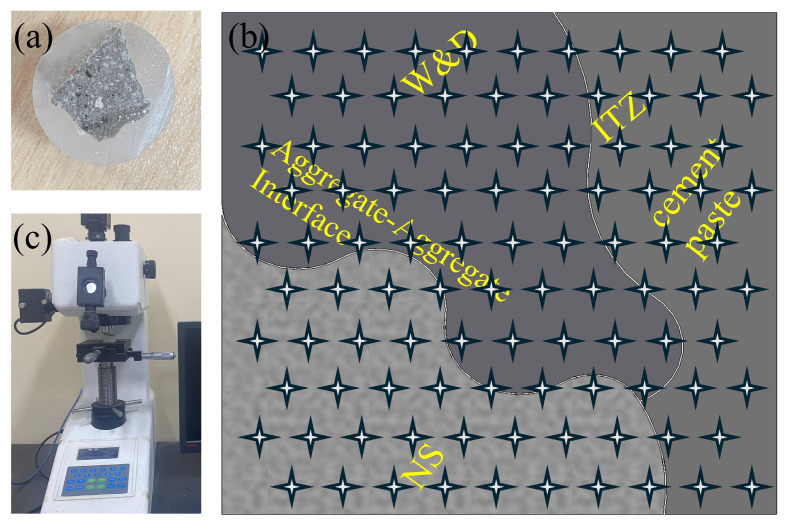
Microhardness test: (**a**) specimen, (**b**) HX-1000T microhardness tester, (**c**) ITZ.

The microhardness and width of the interfacial transition zone (ITZ) of 3D-printed specimens were evaluated using an HX-1000T microhardness tester (Figure 9). To ensure smooth surfaces of the test specimens, the samples were embedded in epoxy resin and then ground and polished with an angle grinder [43]. A total of 100 measurement points near the ITZ were analyzed, with each point subjected to a 100 g load for 10 s. A 10 × 10 grid of microhardness indentations was made across the interface. The measurement points were spaced 20 µm apart. All equipment required for this experiment is listed in Table 6.

## 3. Results and Discussion

### 3.1. Flowability

**Figure 10:**
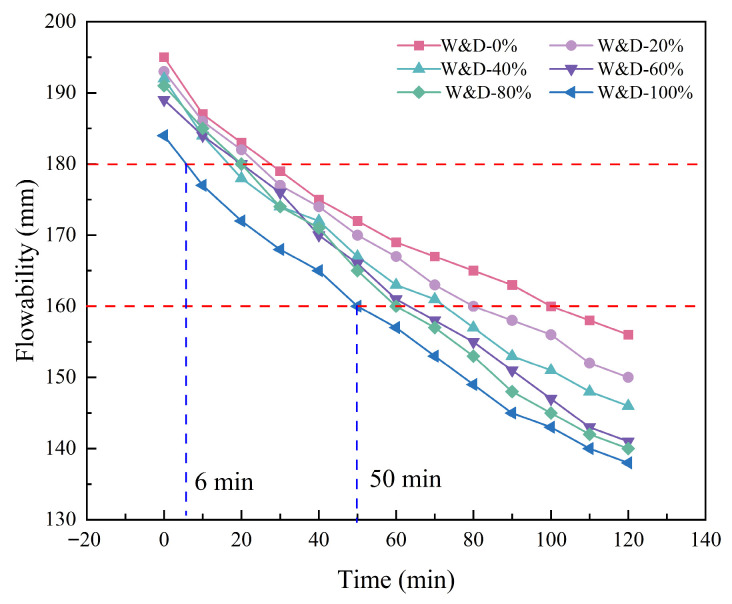
Mortar flowability.

Figure 10 illustrates the flowability of mortar with six different mix proportions. Over time, the spread diameter of the mortar gradually decreased, indicating a reduction in flowability. Based on the morphology and quality of the printed mortar during buildability tests, as well as relevant findings from the literature [44,45], this study identified the suitable flowability range to be between 160 mm and 180 mm, as indicated by the red dashed lines in Figure 10. The spread diameter reached 180 mm at 6 min and decreased to 160 mm by 50 min. All six mixtures maintained flowability within this range for over 44 min. With appropriate water compensation, all mixtures satisfied the required flowability criteria for printing. Specifically, the W&D-0% mixture exhibited an initial spread diameter of 195 mm, which decreased to 156 mm by 120 min, corresponding to a 20% reduction. W&D-20%, W&D-40%, W&D-60%, W&D-80%, and W&D-100% showed initial spread diameters of 193 mm, 192 mm, 189 mm, 190 mm, and 185 mm, respectively, which declined to 150 mm, 146 mm, 141 mm, 140 mm, and 136 mm, reductions of 22.3%, 24.0%, 25.4%, 26.3%, and 26.5%, respectively. The rate of reduction was observed to increase with higher W&D content.

Consistently with previous studies on recycled mortar, the flowability of W&D mortar decreased significantly as the incorporation level increased. With the increase in W&D content, the flowability of fresh mortar exhibited a significant decreasing trend, even when additional water was introduced during mixing. W&D is inherently porous and highly absorbent [46]. During the initial mixing stage, it rapidly absorbs free water, which reduces the lubricating effect of the paste and leads to a decline in initial flowability. Moreover, this rapid water absorption may locally reduce the effective water-to-binder ratio around cement particles, thereby accelerating the early hydration process of cement. These combined effects promote faster setting and hardening of the printed mortar [47].

### 3.2. Buildability and Extrudability

**Figure 11:**
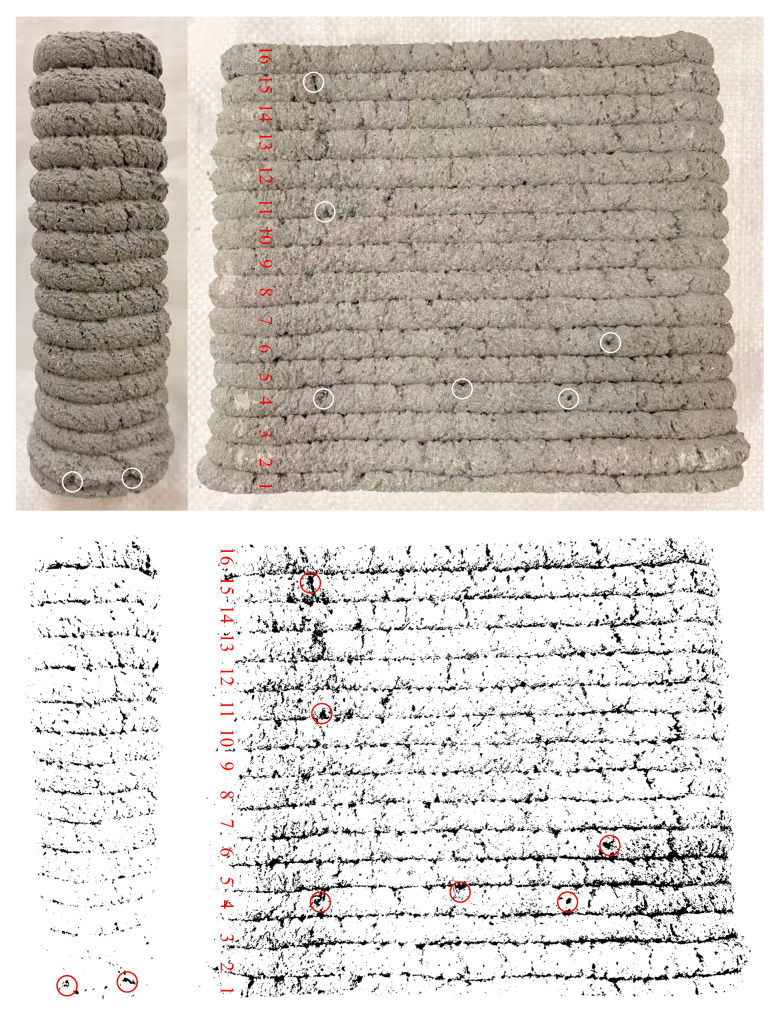
Maximum number of printed layers under W&D-100% conditions.

Figure 11 shows the maximum achievable build height of 3D-printed mortar with the W&D-100% mix proportion. Before setting and hardening, the fresh 3D-printed mortar behaves as a fluid with yield stress, characterized by a certain green yield stress. During the layer-by-layer printing process, the bottom layers bear the cumulative self-weight load from all the freshly deposited layers above. As the printing height increases, the vertical compressive stress on the bottom layers rises linearly. Upon reaching the 17th layer, this compressive stress exceeded the instantaneous yield stress of the material at that moment. At this point, the bottom structure no longer remained in the elastic deformation stage, but underwent irreversible plastic flow and lateral deformation. The resulting geometric changes in the lower structure caused a shift in the center of gravity of the upper layers, introducing additional eccentric bending moments and shear stresses. This local instability propagated rapidly upward, triggering a chain reaction that ultimately led to the instantaneous collapse of the entire structure [48]. The intrinsic imperfections in the printing process also play a significant role in triggering structural instability. During actual printing, minor nozzle vibrations, uneven extrusions can introduce slight initial curvature or geometric eccentricity in the deposited filaments. At lower printing heights, the influence of such defects is negligible. However, as the structure approaches its maximum height and is subjected to extremely high axial loads, it becomes highly sensitive to these minor initial imperfections. Under such conditions, these defects can be drastically amplified, initiating elastic buckling and ultimately leading to sudden lateral collapse of the printed wall.

**Figure 12:**
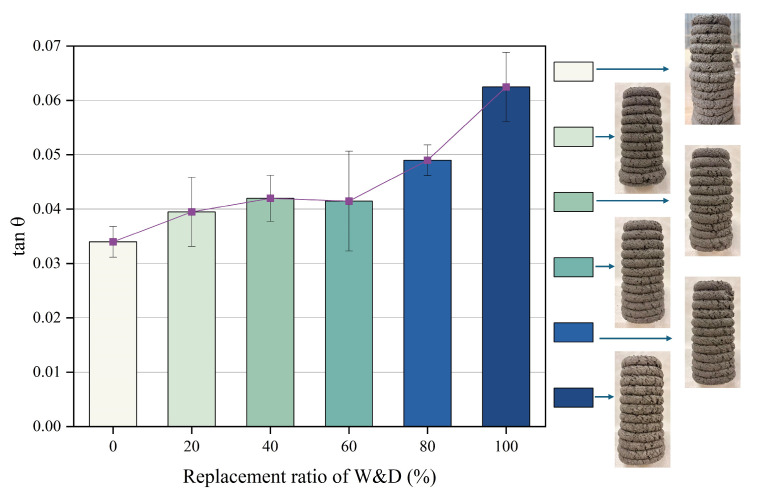
Buildability and tanθ values.

Figure 12 presents the buildability results of 3D-printed mortar with varying W&D replacement ratios. The resistance to plastic deformation and structural instability was evaluated by calculating the tanθ of 12-layer printed specimens. Buildability is one of the essential criteria for printability in 3D printing [49]. The measured tanθ values for the six mortar mixtures were 0.0340, 0.0395, 0.0420, 0.0415, 0.0490, and 0.0625, respectively. All mixtures exhibited satisfactory buildability. As reported in previous research [23], lower values represent excellent buildability. With increasing W&D content, the tanθ values showed an upward trend. Although all mixes met the printing requirements, the buildability of the material gradually decreased. First, the rough, angular morphology of W&D particles increases interparticle friction and mechanical interlocking within the mixture. Secondly, due to the high-water absorption, an appropriate amount of W&D competes for free water in the mortar, thereby increasing the overall viscosity of the mortar. This ensures excellent buildability, while excessive W&D may lead to a slight decrease in buildability.

To ensure smooth extrusion and consistent filament quality, a rotational speed of 1.5 r/s was employed for the extruder [50]. All printed filaments exhibited excellent morphology, characterized by smooth surfaces and continuous deposition without interruption or fracture. Moreover, the deviation between the measured filament width and the target nozzle width was strictly controlled within ±10%, demonstrating favorable controllability and extrusion stability throughout the printing process.

### 3.3. Compressive Strength Analysis

**Figure 13:**
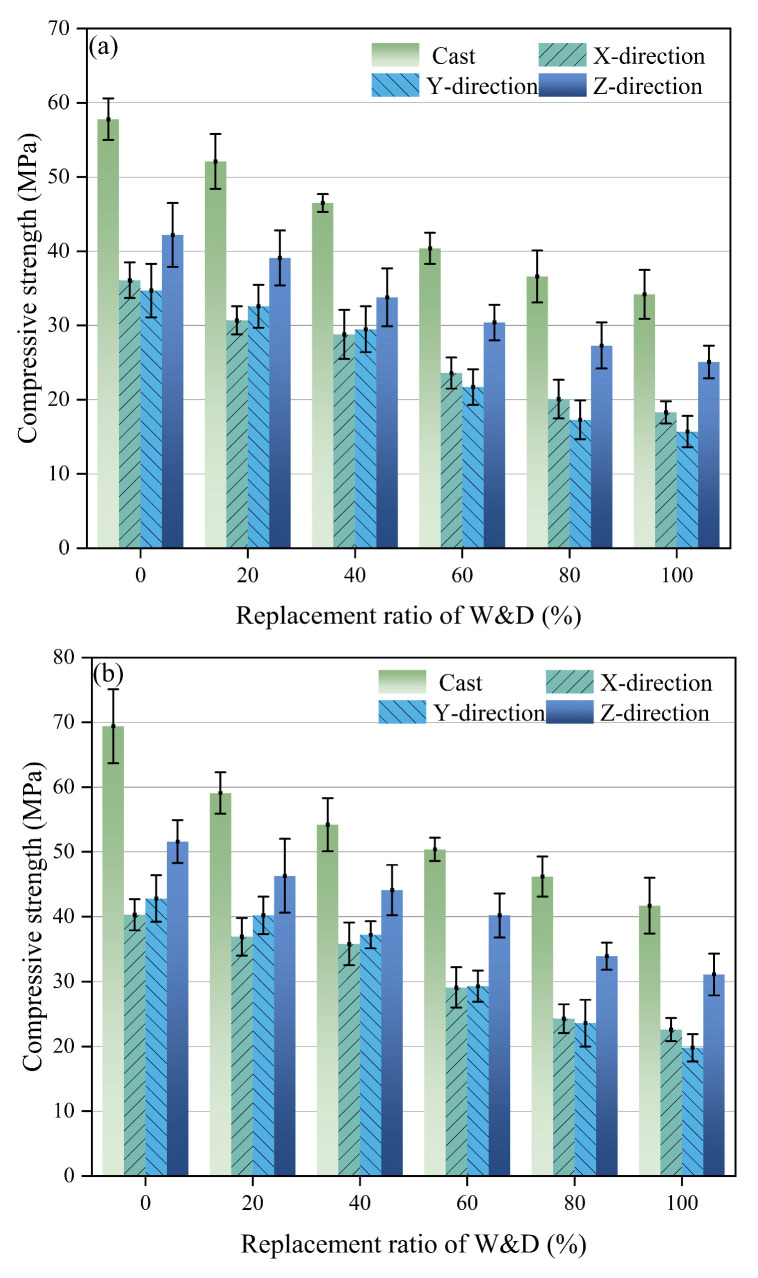
Compressive strength of 3D-printed specimens: (**a**) 7 days, (**b**) 28 days.

The compressive strength of the 3D-printed specimens was tested at 7 and 28 days, including samples with poor buildability to ensure the comprehensiveness and rationality of the mechanical performance evaluation. As shown in Figure 13, the 28-day compressive strength was consistently higher than the 7-day strength, as expected. Additionally, the cast specimens exhibited higher compressive strength than the 3D-printed specimens [51]. With increasing W&D replacement ratio, a decreasing trend in compressive strength was observed. Similarly to the findings of many scholars [52,53], among the 3D-printed mortar specimens, the highest compressive strength was measured in the Z-direction, while strengths in the Y- and X-directions were significantly lower.

Under standard curing conditions, the strength development of mortar is primarily attributed to the continuous hydration of cement. These hydration products filled internal pores, refined the microstructure, and strengthened the ITZ, ultimately resulting in a significant increase in compressive strength from 7 to 28 days. In Figure 13a, the compressive strengths of the cast specimens were 57.8 MPa, 52.1 MPa, 46.5 MPa, 40.4 MPa, 36.6 MPa, and 34.2 MPa, respectively. In comparison, the maximum compressive strengths along the Z-direction of the 3D-printed specimens were 42.2 MPa, 39.1 MPa, 33.8 MPa, 30.5 MPa, 27.3 MPa, and 25.1 MPa, averaging 11.6 MPa lower than those of the cast specimens. The underlying mechanisms of the reduction in compressive strength are systematically discussed in Section 3.8.

Compressive strength is the most important indicator of mechanical properties. Based on this, a three-way analysis of variance (ANOVA) was conducted with reference to previous research [54]. The mix proportion (six levels), curing age (two levels), and loading direction (three levels) were set as fixed factors, and the significance level was defined as α = 0.05.

A three-way ANOVA was conducted using type III sum of squares to evaluate the unique contribution of each factor while controlling all other factors and their interactions. The results are presented in Table 7. All three main factors showed significant effects on compressive strength (*p* < 0.001). Mix proportion had the strongest influence (F = 189.76, η^2^ = 0.739), indicating that the W&D replacement ratio was the dominant factor governing the compressive strength. Curing age (F = 113.58, η^2^ = 0.089) and loading direction (F = 52.43, η^2^ = 0.082) were also significant, confirming that both strength development and anisotropic behavior should be considered in evaluating the mechanical performance of the printed mortar.

In terms of interaction effects, mix proportion × curing age and mix proportion × loading direction were significant (*p* < 0.05), although their effect sizes were relatively small. This indicates that the influence of W&D replacement ratio on compressive strength varied slightly with curing age and loading direction. By contrast, curing age × loading direction and the three-way interaction were not significant (*p* > 0.05), suggesting that no strong higher-order coupling effect was observed. Overall, the ANOVA results confirm that compressive strength was mainly controlled by mix proportion, while curing age and loading direction played secondary, but significant roles.

### 3.4. Flexural Strength Analysis

**Figure 14:**
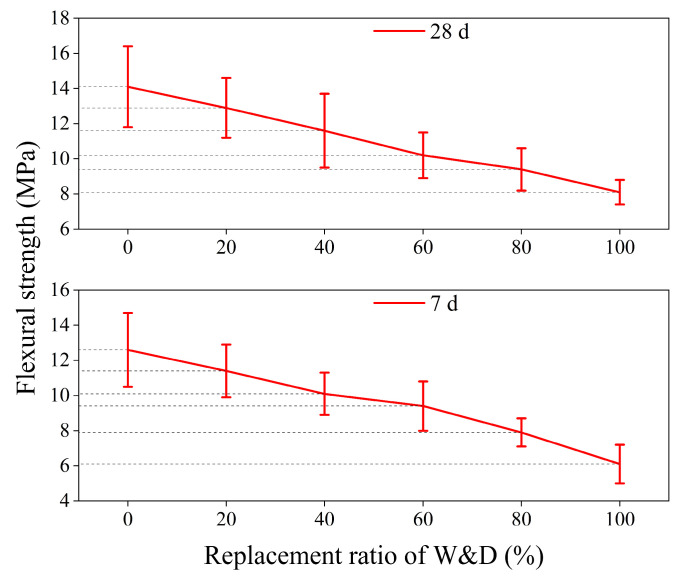
Flexural strength of cast specimens at 7 and 28 days.

As presented in Figure 14, a decreasing trend in flexural strength was observed with increasing W&D replacement ratios. Specifically, the 7-day flexural strengths were recorded as 12.6 MPa, 11.4 MPa, 10.1 MPa, 9.4 MPa, 7.9 MPa, and 6.1 MPa, respectively, representing an overall reduction of 51.6% and an average value of 9.6 MPa. For the 28-day flexural strengths, the values were 14.2 MPa, 12.9 MPa, 11.6 MPa, 10.2 MPa, 9.4 MPa, and 8.1 MPa, respectively, corresponding to a total decrease of 43.0% and an average of 11.1 MPa. Similarly to the behavior observed for compressive strength, the continuous formation and accumulation of hydration products effectively fill internal pores and defects, leading to a denser microstructure and enhanced bonding strength within the ITZ.

**Figure 15:**
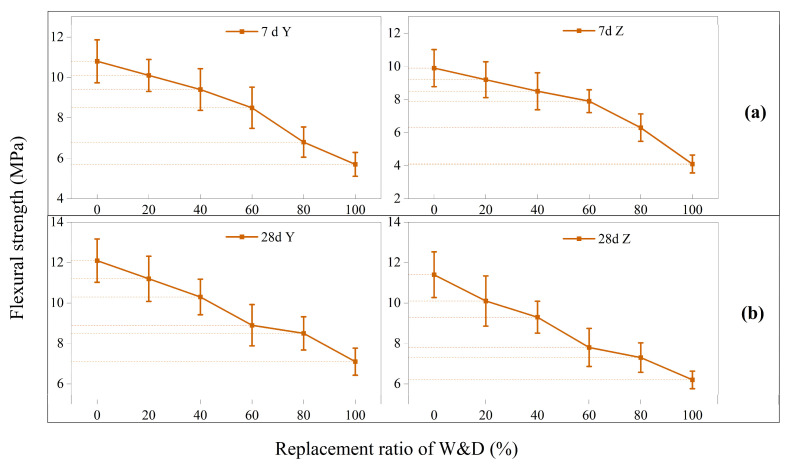
Flexural strength of 3D-printed specimens: (**a**) 7 days, (**b**) 28 days.

In Figure 15, the flexural strength in the Y-direction was higher than that in the Z-direction at both 7 and 28 days. This result is consistent with the findings of other researchers [45,55]. At 7 days, the maximum flexural strength in the Y-direction was 10.8 MPa, the minimum was 5.7 MPa, and the average value was 8.6 MPa. In the Z-direction, the maximum strength was 9.9 MPa, the minimum was 4.1 MPa, and the average was 7.7 MPa. At 28 days, the maximum flexural strength in the Y-direction reached 12.2 MPa, the minimum was 7.1 MPa, and the average was 9.7 MPa. In the Z-direction, the corresponding values were 11.4 MPa (maximum), 6.2 MPa (minimum), and 8.7 MPa (average). The interpretation of the reduction in flexural strength, particularly with respect to the properties of W&D materials, porosity, as well as printing-induced defects, interlayer defects, and anisotropy, is systematically discussed in Section 3.8. This subsection provides an analysis from the perspective of the moment of inertia of a rectangular section.

**Figure 16:**
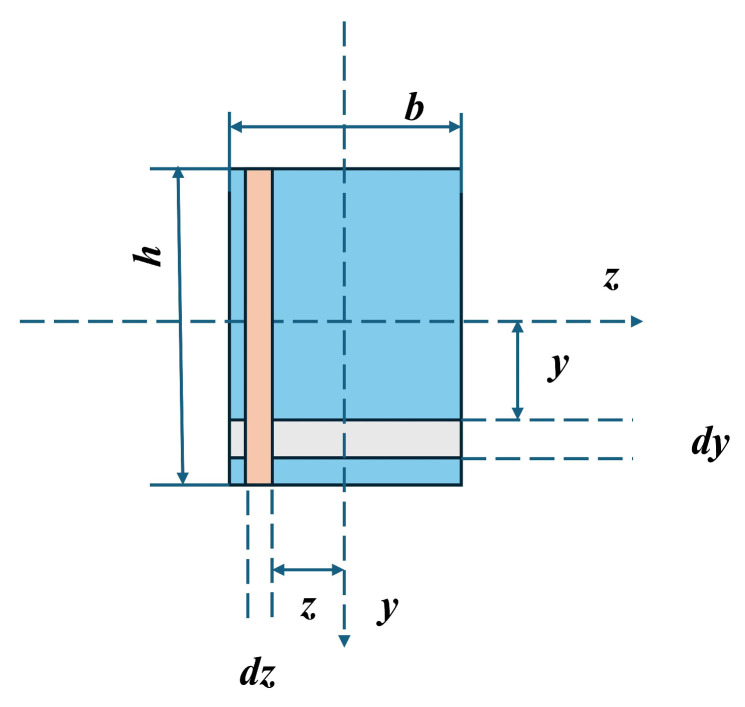
Moment of inertia of rectangular section.

In Figure 16, the interpretation from the perspective of the cross-sectional moment of inertia in structural mechanics can be provided. The moment of inertia is a quantity that reflects the bending resistance characteristics of a section. It is defined as the integral over the entire cross section of each infinitesimal area multiplied by the square of its distance to a specified axis, as indicated in Equations (3)–(6):

(3) $$I_{z}=\int_{A}y^{2}\mathrm{d}A=\frac{1}{12}bh^{3}$$

(4) $$\sigma =\frac{My}{I_{Z}}$$

(5) $$\sigma_{\tau ,max}=\left|\sigma_{c,max}\right|=\frac{\left|M\right|\left|y_{max}\right|}{I_{Z}}=\frac{\left|M\right|}{W_{Z}}$$

(6) $$W_{Z}=\frac{I_{Z}}{\left|y_{max}\right|}=\frac{1}{6}bh^{2}$$

where *b* denotes the cross-sectional width (mm or m), *h* represents the cross-sectional height (mm or m), and *y* and *z* are the neutral axes along the two principal directions. The terms *d_y_* and *d_z_* represent the differential lengths in the two directions. *I_Z_* indicates the moment of inertia of the cross section with respect to the Z-axis, and *W_Z_* is the section modulus about the neutral Z-axis (in mm^3^ or m^3^).

**Figure 17:**
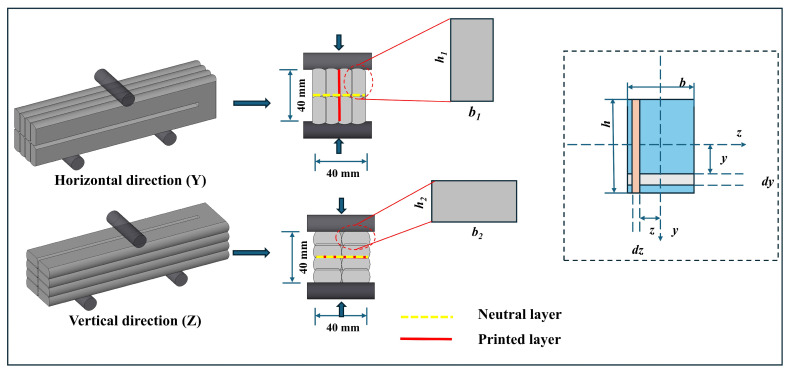
Position of the printed layer with respect to the neutral layer.

As illustrated in Figure 17, the 3D-printed specimens consist of individual printed layers bonded through interlayer adhesion. When a bending load is applied to the specimen, shear stresses develop between the layers, facilitating stress transfer and enabling collaborative load-bearing behavior among them.

Under loading along the Y-direction, the printed layers are oriented perpendicularly to the neutral layer. The layers primarily experience compressive stresses perpendicularly to the interfaces and shear stress parallel to the interfaces. The interlayer bonding can effectively transfer these shear stresses, allowing the layers to deform cooperatively. Consequently, the entire cross section behaves approximately as an integral unit to resist the bending moment, and its effective moment of inertia approaches or equals that of an ideal monolithic specimen. This large effective moment of inertia results in high flexural rigidity. Under loading along the Z-direction, the printed layers are aligned parallel to the neutral layer, representing a typical laminated loading condition. The shear stresses generated by the bending load act in a direction that is most unfavorable for interlayer bond strength, making the structure highly susceptible to delamination. Once interlayer bonding fails, stress transfer between the printed layers is lost, preventing them from working together. At this point, the specimen no longer behaves as an integral “thick beam,” but degrades into multiple independent “thin plates” bending separately.

For *n* layers, each of height *t*, bending independently, the total effective moment of inertia is significantly less than that of a monolithic beam. The moment of inertia of a monolithic beam is proportional to the cube of its height *h*, whereas the sum of the moments of inertia of *n* independent thin layers is only proportional to *h*. Additionally, the section modulus of a single printed filament is twice as high in the Y-direction as in the Z-direction. This explains the notably lower flexural strength observed in the Z-direction.

### 3.5. Interlayer Bond Strength Analysis

**Figure 18:**
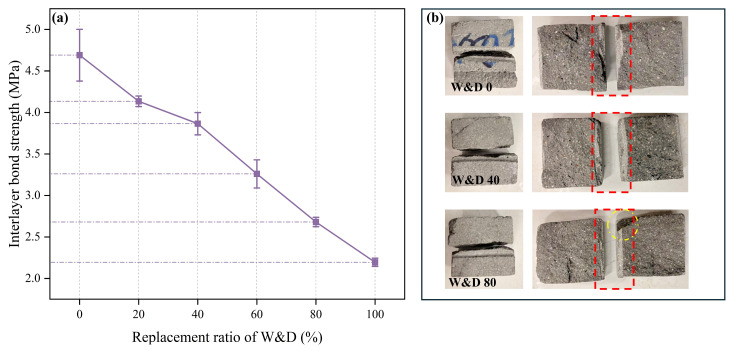
Interlayer bond strength and fracture surface of 3D-printed specimens after 28 days: (**a**,**b**).

As shown in Figure 18a, as the replacement ratio increased from 0% to 100%, the interlayer bond strength dropped sharply from a maximum of 4.72 MPa to a minimum of 2.28 MPa, representing a strength loss of 51.7%. The combined effects of W&D properties and printing defects on interlayer bond strength are further analyzed in Section 3.8.

Figure 18b illustrates the failure modes of 3D-printed mortar layers. The red dashed boxes indicate the grooves cut by the cutting machine. In the W&D-0% specimen, the fracture surface appeared smooth and flat, with clear outlines of the printing paths visible. Aggregate particles were almost entirely pulled out from the interface, leaving regular pits. This indicates that failure occurred exclusively at the bond interface between the mortar matrix and the aggregates, implying that the interlayer bond strength was lower than the strength of the mortar matrix itself [56]. As the W&D content increased, the fracture surfaces became generally rougher. Due to non-uniform stress distribution, partial shear failure of W&D particles was observed (as indicated by the yellow dashed areas). Given the inherently lower strength of W&D, as the interfacial bond strength decreased with increasing porosity, the failure mode transitioned from interfacial bond failure to a mixed mode involving both failure of the aggregate itself and interfacial bond failure. SEM images and MIP results further confirmed that the interfacial zone was predominantly composed of loose and porous hydration products accompanied by numerous microcracks. These factors contributed to the significant degradation in interlayer bond strength [57].

### 3.6. SEM Analysis

**Figure 19:**
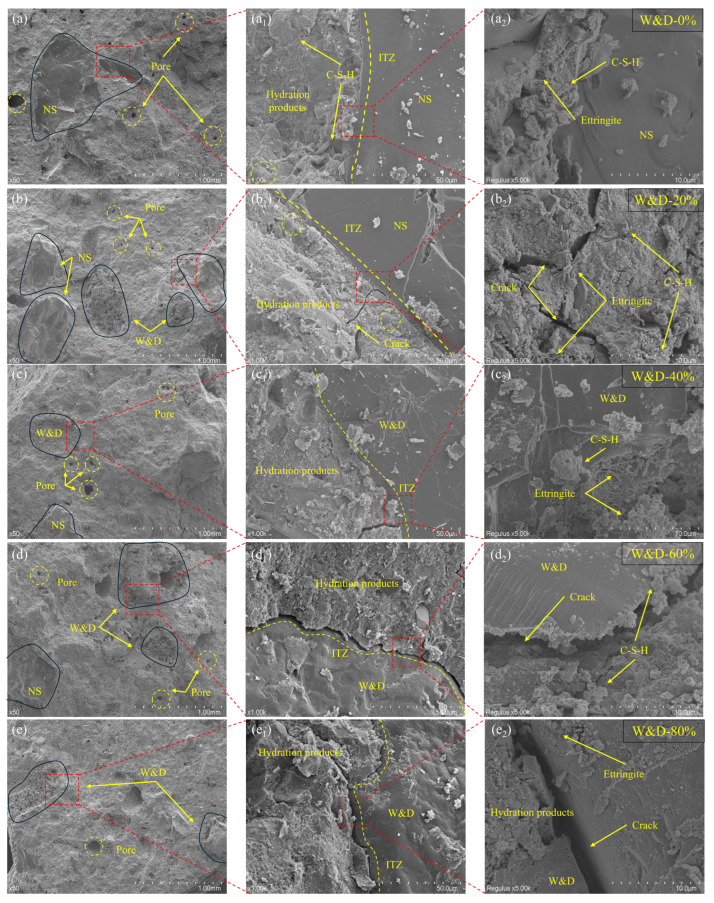

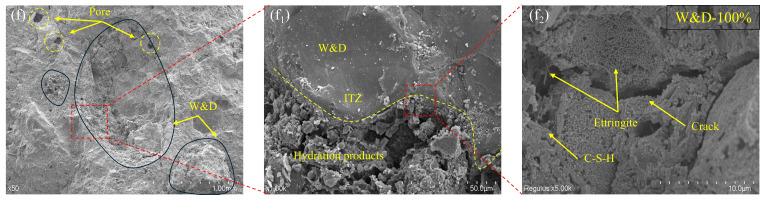
SEM images of the 3D-printed specimens after 28 days: (**a**–**f**) 50×, (**a_1_**–**f_1_**) 1000×, (**a_2_**–**f_2_**) 5000×.

At 50× magnification, as shown in Figure 19a, aggregate particles were clearly observed, exhibiting a generally dense and uniform surface morphology. Only a few isolated capillary pores were present, with no significant continuous microcracks observed. As the W&D content increased, as shown in Figure 19f, a greater number of demolition waste aggregates became visible. These aggregates displayed numerous needle-like pores on their surfaces, and the fracture surface exhibited a loose, porous structural characteristic.

At 1000× magnification, as shown in Figure 19(a_1_), the ITZ between natural sand particles and the cement paste appeared narrow and densely structured. This region was predominantly composed of interwoven C-S-H, which exhibited continuity with the bulk paste matrix. No significant enrichment of CH crystals or microcracks was observed. In Figure 19(b_1_–d_1_), a slight increase in ITZ width is noted. A layer of loose, flocculent C-S-H was visible on the surface of the aggregate particles. Although some W&D particles showed good bonding with the paste, intermittent microcracks had begun to appear in the fresh mortar portion of the ITZ. In Figure 19(e_1_,f_1_), the ITZ width increases markedly, forming a continuous, loose, and porous band-like area. This zone was filled with loose, flocculent C-S-H, and a substantial amount of platy CH crystals were densely concentrated along the aggregate surface. Microcracks and pores interconnected and propagated along this weak zone, leading to noticeable debonding between the aggregates and the paste. Consequently, with increasing W&D content, the ITZ progressively transitioned from the narrow and dense morphology observed in W&D-0% to a wider and more porous structure in W&D-100%.

At a higher magnification of 5000×, as shown in Figure 19(a_2_), the paste in the ITZ was tightly bonded to the aggregate. Dense C-S-H were interwoven, forming a continuous and robust three-dimensional network that encapsulated and firmly bound the cement particles. No continuous defects or microcracks were observed. The surface of the natural sand aggregate was densely covered by C-S-H, with ettringite embedded within the structure. In Figure 19(b_2_–d_2_), significant degradation of the ITZ was evident. First, the amount of dense C-S-H decreased, being replaced by a large quantity of loose, granular, and flocculent C-S-H, which exhibited markedly reduced structural strength and bonding capacity. Second, microcracks and pores began to appear and interconnect between the W&D surfaces and the flocculent C-S-H. In Figure 19(e_2_,f_2_), a continuous microcrack is clearly visible along the aggregate surface, separating the aggregate from the paste. SEM analysis clearly revealed the microstructural mechanisms underlying the decline in macroscopic properties with increasing W&D content: the high-water absorption of W&D led to insufficient space for complete hydration, resulting in a porous structure, and a wide and weak ITZ formed around the W&D. This was manifested as a reduction in dense C-S-H and an increase in loose hydration products, voids, and cracks [53].

### 3.7. MIP Analysis

Based on the relevant literature [58,59], pores are classified into five categories according to their diameter—<10 nm, 10 nm–100 nm, 100 nm–1 μm, 1 μm–10 μm, and >10 μm—which correspond to gel pores, medium pores, medium-capillary pores, large-capillary pores, and macropores, respectively. An additional boundary of 100 μm was introduced in the context of 3D printing. This extended classification includes <10 nm, 10 nm–100 nm, 100 nm–1 μm, 1 μm–10 μm, 10 μm–100 μm, and >100 μm, referring to gel pores, medium pores, medium-capillary pores, large-capillary pores, coarse pores, and ultralarge pores, respectively.

**Figure 20:**
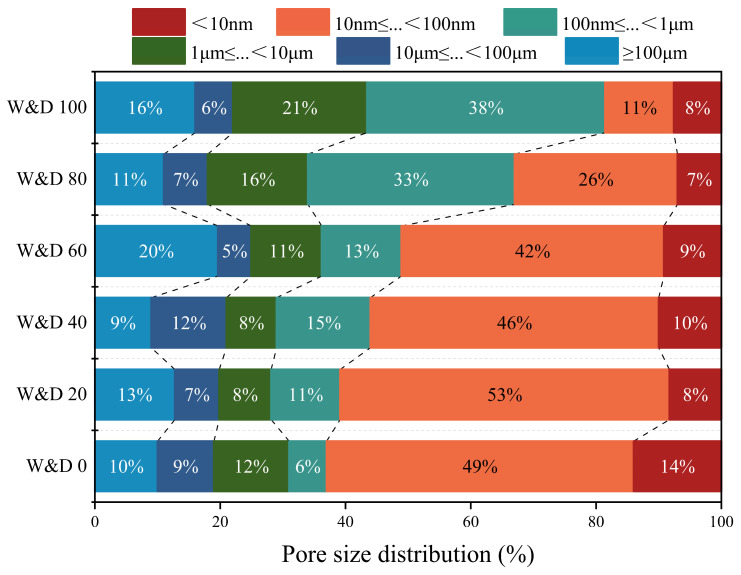
Pore size distribution of the 3D-printed specimens after 28 days.

As shown in Table 8, the total porosity demonstrated a consistently increasing trend, rising from 14.7443% to 27.5903%. In Figure 20, the analysis indicates that as the W&D replacement ratio increased from 0% to 100%, the pore size distribution shifted toward the harmful capillary pore range, primarily toward medium-capillary pores. The proportion of gel pores and medium pores decreased from 63% to 19%, a reduction of 44%, while the proportion of medium-capillary pores increased from 6% to 38%, representing a critical range of pore structure deterioration. This increase in harmful pores can be attributed to several factors. The rough surface and complex morphology of W&D were observed to exacerbate the degradation of the ITZ, introducing numerous weak bonding zones and gaps between the new paste and the old particles. To maintain adequate fluidity of the mortar at a high replacement ratio, additional water was required. This excess water not only evaporated after hardening, leaving behind voids, but also increased the spacing between cement particles, leading to a looser microstructure of the cementitious matrix. Consequently, the medium-capillary pores formed during early hydration could not be effectively filled and were preserved in the hardened mortar [60,61].

### 3.8. Relationship Between Mechanical Properties, Porosity, and Printing-Induced Defects

**Figure 21:**
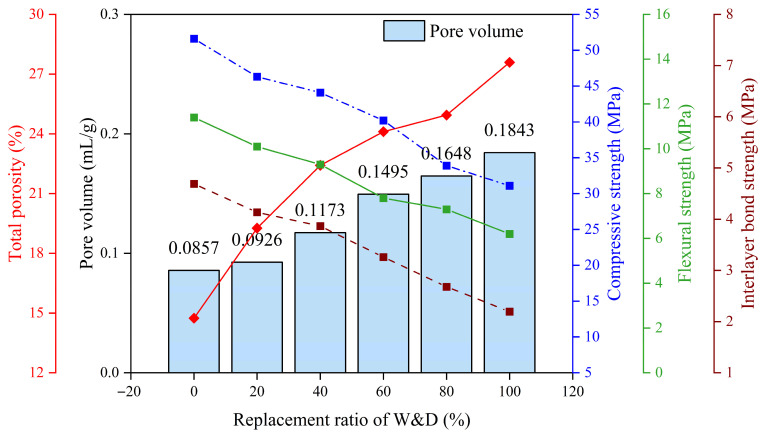
Relationship between mechanical properties and porosity.

Figure 21 systematically illustrates the variation patterns of total porosity, pore volume, compressive strength, flexural strength, and interlayer bond strength of mortar after 28 days of curing under different W&D substitution rates. Analysis of the pore volume trend reveals that both total pore volume and total porosity continuously increase with higher W&D content, indicating that the incorporation of W&D significantly enhances the porous structure of the material by introducing more and larger pores as well as weak interfaces, which is the primary reason for the decline in mechanical properties with increasing substitution.

#### 3.8.1. Influence of W&D Material Properties

Mechanical properties are primarily governed by mechanical interlocking and chemical bonding at the interface between successively printed mortar layers [62,63]. NS particles, with their smooth surfaces and stable chemical properties, facilitate the formation of a relatively uniform ITZ with fresh cement paste. In contrast, W&D exhibits rough surfaces and angular shapes and is often covered with porous old cement paste. At the interface, the new cement paste bonds not only with the aggregate itself but more critically with the aged cement paste adhered to the W&D particles. This results in insufficient cement hydration and the formation of a weaker and more porous hydration product structure, thereby compromising the strength. To achieve flowability comparable to that of natural mortar, the mixing water content had to be increased. The excess free water, not consumed by cement hydration, eventually evaporates, leaving more pores and defects in the hardened mortar, which also contributes to the reduction in mechanical properties.

#### 3.8.2. Influence of Interlayer Defects

In all mix proportions, the cast specimens consistently exhibited higher mechanical properties than the 3D-printed specimens. This is attributed to the absence of vibration during the 3D printing process, which can lead to defects such as interlayer gaps and air voids, thereby weakening the printed specimens [64]. Furthermore, higher W&D content was found to adversely affect the rheological properties of the mortar. The extruded filaments exhibited rougher surfaces and were more prone to microcracking. This reduction in effective contact area contributed to interlayer failure under loads [65].

#### 3.8.3. Influence of Anisotropy

The 3D-printed specimens displayed significant anisotropy in mechanical properties [66]. For compressive strength and flexural strength, the Z-direction strength was markedly higher than those in the X- and Y-directions. When loaded along the Z-direction, the stress is mainly borne by the mortar matrix, leading to crushing failure of the material and thus the highest strength. In contrast, when loaded along the X- or Y-direction, failure is governed by the relatively weak interlayer bonding, manifesting as delamination or shear slip along the interfaces.

#### 3.8.4. Influence of Printing-Induced Defects

The inherent defects of the 3D printing process (such as uneven extrusion, interlayer pauses, and nozzle vibration), together with the characteristics of W&D materials, collectively lead to a significant decline in mechanical performance at high replacement ratios.

### 3.9. Microhardness Analysis

**Figure 22:**
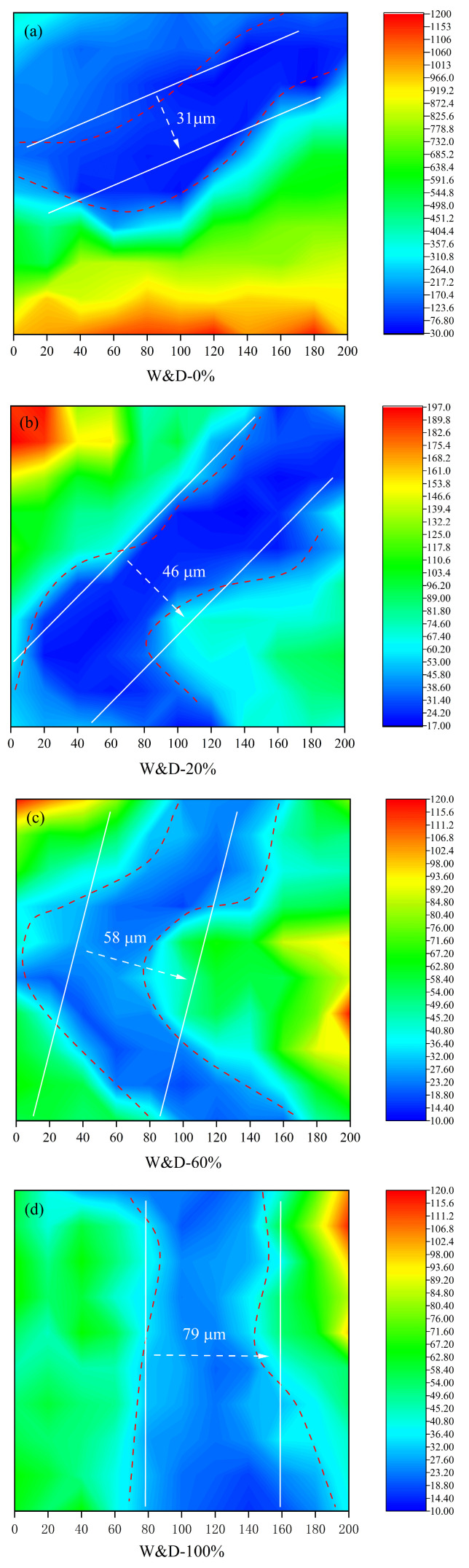
Microhardness test results of the 3D-printed specimens after 28 days: (**a**) W&D-0%, (**b**) W&D-20%, (**c**) W&D-60%, (**d**) W&D-100%.

In Figure 22, the width of the ITZ is observed to increase significantly with higher incorporation of W&D. In this study, the boundaries of the ITZ are defined based on differences in microhardness among the aggregate, ITZ, and mortar matrix, with the average width being used as the quantification indicator [67]. Test results indicate that as the W&D replacement ratio increases from 0% to 20%, 60%, and 100%, the average ITZ width is increased from 31 μm to 46 μm, 58 μm, and finally to 79 μm. The marked deterioration of the ITZ is primarily attributed to the inherent physical characteristics of the W&D. These aggregates are characterized by rough surfaces and high internal porosity, leading to intense absorption of free water from the cement paste during mixing. Consequently, a sharp reduction in the local effective water–cement ratio is induced in the aggregate-paste interfacial region. The insufficient water availability severely hinders the formation of dense C-S-H, and instead, a large volume of loose and porous hydration products is formed. This mechanism is consistent with SEM observations: with increasing W&D content, a broad and porous microscopic weak zone is gradually developed within the ITZ region [68]. The formation and propagation of this weak structure are identified as the fundamental cause of the notable decline in macroscopic mechanical properties as the W&D content is raised [69].

### 3.10. Environmental Impacts

Life cycle assessment (LCA) has been widely applied to evaluate the environmental impacts and resource consumption associated with a product throughout its entire life cycle [35,70]. In this study, global warming potential (GWP) was selected as the main indicator to compare the carbon emissions of mortars containing different replacement ratios of W&D. To maintain consistency with the material inventory listed in Table 4, the functional unit was defined as 1 kg of fresh mortar, and the results are expressed in kg CO_2_-eq/kg mortar. The system boundary was limited to the cradle-to-gate stage, including raw material production, waste processing, and transportation to the mixing and printing site.

Since all mix proportions were prepared using the same printing parameters, the environmental contribution from raw material production was considered to be far greater than any differences in printing energy consumption. Furthermore, given that the curing and end-of-life stages were assumed to be identical for all mixtures during the exploratory phase, the printing energy, raw material production, transportation, and end-of-life processes were not taken into account in this study. Therefore, a cradle-to-gate approach was considered appropriate for isolating the influence of raw material substitution on GWP.

The emission factors of cement, silica fume, river sand, W&D, sodium gluconate, superplasticizer, and water were obtained from published literature, as listed in Table 9. The total GWP of each mortar mixture was calculated using the following formula:

(7) $$Total\;GWP=\sum_{i}^{n}M_{i}C_{i}$$

where *i* denotes the type of raw material, *M_i_* is the mass fraction of raw material *i* in 1 kg of mortar, and *C_i_* is the emission factor (EF) of raw material *i* (kg CO_2_-eq/kg).

In the revised calculation, the material masses in Table 5 were first normalized by the total mass of each mixture, and the GWP values were then expressed uniformly in kg CO_2_-eq/kg mortar. The detailed content is shown in Table 9.

As shown in Table 10, the GWP of the mortar decreased gradually with increasing W&D replacement ratio. The calculated values for W&D-0%, W&D-20%, W&D-40%, W&D-60%, W&D-80%, and W&D-100% were 0.4043, 0.3953, 0.3904, 0.3854, 0.3822, and 0.3800 kg CO_2_-eq/kg mortar, respectively. Compared with the control group, the W&D-100% mixture showed an overall reduction of approximately 5.99% in GWP. This trend is mainly attributed to the progressive reduction in natural sand consumption and the low emission factor assigned to recycled W&D aggregate. Although the additional mixing water increased slightly with increasing W&D content, its contribution to the total GWP remained negligible because the emission factor of water was very low.

To evaluate the robustness of the environmental assessment, a simple sensitivity analysis was conducted by varying the emission factors of the two key parameters, namely cement and recycled W&D aggregate, by ±10%. The results are summarized in Table 11. When the cement emission factor was varied by ±10%, the total GWP of all mixtures changed by approximately ±9.9%, confirming that cement remained the dominant contributor to the overall carbon footprint. In contrast, varying the emission factor of W&D by ±10% caused only negligible changes in the total GWP, with the maximum variation being about ±0.01% for the W&D-100% mixture. More importantly, the overall ranking of the mixtures remained unchanged under all scenarios, and the GWP still decreased monotonically with increasing W&D replacement ratio. This indicates that the comparative environmental conclusion of the present study is robust within a reasonable uncertainty range.

Several sources of uncertainty should be acknowledged in the present LCA. First, the EF was collected from published literature, which may vary depending on region, production technology, and database assumptions. Second, the recycled W&D aggregate is inherently heterogeneous, and its processing burden may change with impurity content, washing efficiency, and crushing intensity. Third, this study excluded on-site printing energy, curing, use phase, and end of life, which may affect the absolute environmental burden of the system. Therefore, the current LCA results should be interpreted as a comparative material-stage assessment rather than a complete whole-life evaluation.

Increasing the W&D replacement ratio reduced the carbon footprint per unit mass of mortar, but it also led to a clear deterioration in mechanical properties and pore structure. Maximizing the utilization of W&D is key to achieving efficient solid waste resource utilization. At a replacement ratio of 80%, the compressive strength of the mortar exceeds 33 MPa and its flexural strength exceeds 6.5 MPa. In this study, considering factors such as printability, mechanical properties, and carbon emissions, 80% is identified as the optimal replacement ratio.

## 4. Conclusions

Based on the recycling of construction and demolition waste into usable W&D recycled fine aggregate and its application in 3D-printed mortar, this study investigated the effects of different W&D replacement ratios on the printability, mechanical properties, and environmental carbon emissions of 3D-printed cement mortar. The main conclusions are as follows.

1. The 3D-printed mortar incorporating W&D exhibited excellent buildability. However, as the content of W&D increased, the flowability and extrudability were reduced, even with additional water.

2. With the increase in the replacement ratio of W&D, the compressive strength, flexural strength, and interlayer bonding strength of both cast and 3D-printed specimens decreased. The compressive strength of the printed specimens was lower than that of the cast specimens. Regarding the anisotropy of compressive strength, the highest strength was observed in the Z-direction, while the strength in the X- and Y-directions was relatively lower. After 28 days of curing, the compressive strength of the W&D-80% nearly reached 35 MPa, showing a decrease of 17.6 MPa compared to the 0% reference group.

3. As the replacement ratio of W&D increased, the pores and cracks on the surface of the 3D-printed specimens exhibited a distinct gradient distribution, primarily concentrated in the bottom one to five layers. For the W&D-100%, the maximum achievable build height of the 3D-printed mortar was 16 layers, with collapse occurring at the 17th layer due to defects in the bottom layers.

4. XRF, TG, and XRD results indicated that the calcium content in the W&D was as high as 52%, primarily present in C-S-H, Ca (OH)_2_, and calcium carbonate. Combined with SEM images, the W&D particles exhibited a loose, porous, and angular morphology with a rough surface texture, exhibiting high water absorption. Microhardness tests revealed that the interfacial transition zone was significantly weakened with an increase in the W&D content, with the maximum ITZ width reaching 79 μm.

5. MIP results showed that as the replacement ratio of W&D increased from 0% to 100%, the total porosity of the 3D-printed specimens significantly increased from 14.7433% to 27.5903%. The proportion of gel pores and mesopores decreased by 44%, while the proportion of medium-capillary pores increased from 6% to 38%, which is the primary reason for the decline in mechanical properties with increasing substitution.

6. As the W&D replacement ratio increased from 0% to 100%, the total GWP decreased from 0.4043 kg CO_2_-eq/kg mortar to 0.3800 kg CO_2_-eq/kg mortar. Considering both environmental performance and mechanical properties, an 80% replacement ratio provided a relatively balanced option for practical application.

Future research should further advance this topic from three perspectives. First, more dedicated efforts should be made to establish a quantitative correlation between anisotropic mechanical behavior and interface characteristics, particularly in terms of interlayer bond strength, ITZ evolution, and microstructural heterogeneity. Second, the environmental assessment should be broadened by incorporating scenario-based analyses of transport distance, printing electricity consumption, curing regime, and end-of-life recycling pathways so as to improve the robustness and practical relevance of the evaluation. Third, to enhance the engineering applicability of high-replacement W&D mortar, the ITZ structure should be further improved through the incorporation of microsilica or nanomaterials, and the resulting effects on printability, mechanical performance, and durability should be systematically investigated.

## Figures and Tables

**Table 1:** Chemical compositions of cement.

Chemical Composition	CaO	SiO_2_	Al_2_O_3_	Fe_2_O_3_	MgO	K_2_O	SO_3_	Others
Mass fraction (%)	71.63	12.51	4.32	3.32	1.23	0.75	2.06	4.18

**Table 2:** Physical properties of fine aggregate.

Fine Aggregate	Apparent Density (kg/m^3^)	Water Absorption (%)	Fineness Modulus	Crushing Value (%)
NS	2580.0	2.9	2.43	14.5
W&D	2039.5	22.4	3.57	36.2

**Table 3:** Chemical composition of silica fume.

Chemical Composition	CaO	SiO_2_	Al_2_O_3_	Fe_2_O_3_	SO_3_	Others
Mass fraction (%)	1.43	96.22	0.71	0.25	0.31	1.08

**Table 4:** List of material information.

Materials	Manufacturer	City	Country
Cement	Changzhou China Railway Urban Construction Components Co., Ltd.	Changzhou	China
NS	Jiangsu Lvhe Environmental Technology Co., Ltd.	Changzhou	China
W&D	Jiangsu Lvhe Environmental Technology Co., Ltd.	Changzhou	China
SP	Jiangsu Sobute New Materials Co., Ltd.	Nanjing	China
SG	Xilong Scientific Co., Ltd.	Shantou	China
SF	Henan Borun Casting Materials Co., Ltd.	Gongyi	China

**Table 5:** Mixture proportion of raw materials for 3D printing by mass ratio.

Mix	Cement (g)	SF (g)	NS (g)	W&D (g)	SG (g)	SP (g)	Water-o (g)	Water-a (g)
W&D-0%	1000	50	1000	0	1.2	1.0	350	0
W&D-20%	1000	50	800	200	1.2	1.0	400	1
W&D-40%	1000	50	600	400	1.2	1.0	400	28
W&D-60%	1000	50	400	600	1.2	1.0	400	57
W&D-80%	1000	50	200	800	1.2	1.0	400	74
W&D-100%	1000	50	0	1000	1.2	1.0	400	85

Note: Water-o denotes the base water usage, and water-a indicates the additional water mass (g).

**Table 6:** Equipment list.

Equipment Name	Model	Manufacturer	City	Country
Laboratory-grade concrete (mortar) 3D printer	GL-3DPRT-L	Hangzhou Guanli Intelligent Technology Co., Ltd.	Hangzhou	China
Cement mortar mixer	JJ-5	Wuxi Jianyi Experiment Instrument Co., Ltd.	Wuxi	China
Apparatus of fluidity of cement mortar	NLD-3	Wuxi Jianyi Experiment Instrument Co., Ltd.	Wuxi	China
Microhardness tester	HX-1000T	Shanghai Yuguang Instrument Co., Ltd.	Shanghai	China
Ultrahigh-resolution field-emission scanning electron microscope	Regulus 8100	Hitachi High-Tech Corporation	Tokyo	Japan
Smart X-ray diffractometer	SmartLab 9kW	Rigaku Corporation	Tokyo	Japan
Wavelength-dispersive X-ray fluorescence spectrometer	S8 TIGER	Bruker Corporation	Karlsruhe	Germany
Automatic mercury intrusion porosimeter	AutoPore IV 9510	Micromeritics Instrument Corporation	Norcross	USA
Microcomputer-controlled electrohydraulic servo universal testing machine	WAW-600C	Jinan Shijin Instrument Co., Ltd.	Jinan	China

**Table 7:** Three-way ANOVA results (type III sum of squares) for compressive strength.

Source of Variation	SS	df	MS	F-Value	*p*-Value	η^2^
Mix proportion (a)	7052.89	5	1410.58	189.76	<0.001	0.739
Curing age (b)	844.37	1	844.37	113.58	<0.001	0.089
Loading direction (c)	779.44	2	389.72	52.43	<0.001	0.082
a × b	89.46	5	17.89	2.41	0.042	0.0094
a × c	156.32	10	15.63	2.1	0.031	0.0164
b × c	12.15	2	6.08	0.82	0.444	0.0013
a × b × c	68.54	10	6.85	0.92	0.516	0.0072
Residual	535.2	72	7.43	/	/	/

SS is the sum of squares, df is the degrees of freedom, and MS is the mean square.

**Table 8:** Total porosity of W&D mortar with six mixed proportions.

Total Porosity	W&D-0%	W&D-20%	W&D-40%	W&D-60%	W&D-80%	W&D-100%
%	14.7443	19.2638	22.4192	24.1117	24.9471	27.5903

**Table 9:** Normalized mass fractions of raw materials in 1 kg of mortar (kg).

Mix Proportion	Cement	SF	NS	W&D	SG	SP
W&D-0%	0.4163	0.0208	0.4163	0	0.0005	0.0004
W&D-20%	0.4076	0.0204	0.3261	0.0815	0.0005	0.0004
W&D-40%	0.4032	0.0202	0.2419	0.1613	0.0005	0.0004
W&D-60%	0.3985	0.0199	0.1594	0.2391	0.0005	0.0004
W&D-80%	0.3959	0.0198	0.0792	0.3167	0.0005	0.0004
W&D-100%	0.3941	0.0197	0	0.3941	0.0005	0.0004

**Table 10:** Emission factors of raw materials and carbon emission contributions of each mix proportion (kg CO_2_-eq/kg mortar).

Raw Material	CO_2_ Emission (kg CO_2_-eq/kg)	Reference	W&D-0%	W&D-20%	W&D-40%	W&D-60%	W&D-80%	W&D-100%
Cement	0.9600	[71]	0.399634	0.391326	0.387066	0.382592	0.380017	0.37837
SF	0.0170	[72]	0.000354	0.000346	0.000343	0.000339	0.000336	0.000335
River sand	0.0080	[73]	0.00333	0.002609	0.001935	0.001275	0.000633	0
W&D	0.0010	[74]	0	0.000082	0.000161	0.000239	0.000317	0.000394
SG	1.2000	[75]	0.000599	0.000587	0.000581	0.000574	0.00057	0.000568
SP	0.7200	[76]	0.0003	0.000293	0.00029	0.000287	0.000285	0.000284
Water	0.0003	[77]	0.000044	0.000049	0.000052	0.000055	0.000056	0.000057
Total	/	/	0.404261	0.395292	0.390428	0.385361	0.382215	0.380008

**Table 11:** Sensitivity analysis of GWP under ±10% variation in key emission factors (kg CO_2_-eq/kg mortar).

Scenario	W&D-0%	W&D-20%	W&D-40%	W&D-60%	W&D-80%	W&D-100%
Baseline	0.4043	0.3953	0.3904	0.3854	0.3822	0.3800
Cement EF −10%	0.3643	0.3562	0.3517	0.3471	0.3442	0.3422
Cement EF +10%	0.4442	0.4344	0.4291	0.4236	0.4202	0.4178
W&D EF −10%	0.4043	0.3953	0.3904	0.3853	0.3822	0.3800
W&D EF +10%	0.4043	0.3953	0.3904	0.3854	0.3822	0.3800

## Data Availability

The original contributions presented in this study are included in the article. Further inquiries can be directed to the corresponding author.
